# Supplementary material for: Hyperpolarized 13C-Pyruvate Metabolism as a Surrogate for Tumor Grade and Poor Outcome in Renal Cell Carcinoma—A Proof of Principle Study
Source: Cancers (Basel). 2022 Jan 11;14(2):335. doi: 10.3390/cancers14020335 (PMC8773685; doi:10.3390/cancers14020335)
Supplement: Supplementary file 1 [file cancers-14-00335-s001.zip › cancers-1488329-supplementary.pdf]

cancers

MDPI

Supplementary Materials

Hyperpolarized ^{13}C-pyruvate metabolism as a surrogate for tumor grade and poor outcome in renal cell carcinoma – a proof of principle study

Stephan Ursprung, Ramona Woitek, Mary A. McLean, Andrew N. Priest, Mireia Crispin-Ortuzar, Cara R. Brodie, Andrew B. Gill, Marcel Gehrung, Lucian Beer, Antony C. P. Riddick, Johanna Field-Rayner, James T. Grist, Surrin S. Deen, Frank Riemer, Joshua D. Kaggie, Fulvio Zaccagna, Joao A. G. Duarte, Matthew J. Locke, Amy Frary, Tevita F. Aho, James N. Armitage, Ruth Casey, Iosif A. Mendichovszky, Sarah J. Welsh, Tristan Barrett, Martin J. Graves, Tim Eisen, Thomas J. Mitchell, Anne Y. Warren, Kevin M. Brindle, Evis Sala, Grant D. Stewart and Ferdia A. Gallagher

Supplementary Methods S1

Hyperpolarized [1-^{13}C]pyruvate sample preparation

^{13}C-labeled pyruvate was hyperpolarized for approximately ~3 h at 5 T and ~0.8 K using a clinical hyperpolarizer (SPINlab, Research Circle Technology, Niskayuna, NY).

The sample contained 1.47 g of [1-^{13}C]pyruvic acid (Sigma Aldrich, St Louis, Missouri, USA) and 15 mM of an electron paramagnetic agent (EPA, AH111501, Syncom, Groningen, Netherlands) and was irradiated with microwaves at a frequency of 139 GHz. The sample was rapidly dissolved in 38 ml of superheated sterile water and filtered to reduce the EPA concentration. The filtered formulation was neutralized with a buffer solution (19 ml sterile water and 17.5 ± 0.5 g NaOH/Tris/EDTA (2.4%, 4.03% and 0.033% w/v respectively, Royal Free Hospital, London) at pH 13.4). Sample pH (6.5–8.2, temperature (25–37°C), pyruvate (210 – 280 mM) and EPA concentrations (≤3 µM) were verified in the SPINlab quality control (QC) module (acceptable limits for injection in brackets). After filtration (0.2 µm; ZenPure, Manassas, VA, USA), 0.4 ml of polarized pyruvate solution per kg of patient body weight was injected into a venous catheter in the antecubital fossa at 5 ml/s, followed by a 25 ml saline flush (power injector: MedRad Spectris Solaris EP MR Injection System, Warrendale, Pennsylvania, USA). Quality control parameters of the injected pyruvate solutions are summarized in Table S3.

Proton MRI Technique

Fat-suppressed coronal T_1-weighted (T_1w) images were acquired using a breath-hold 3D Dixon sequence (GE-implementation: LAVA-Flex) with the following parameters: TE: 1.1/2.2 ms; TR: 3.8 ms; flip angle 10°; field of view (FoV) 40 cm × 40 cm; slice thickness 4 mm; acquisition (reconstruction) matrix 224×224×40 (256×256×80); receiver bandwidth ±143 kHz; parallel imaging (ARC) factor 1.5; acquisition time 17 s.

Coronal T_2-weighted (T_2w) images were acquired using a respiratory navigator-triggered 3D fast spin-echo sequence with inner volume excitation (GE implementation: FOCUS/HyperCUBE), using fat suppression and the following parameters: TE: ~100 ms; TR: 1 respiratory cycle; FoV 40 cm × 36 cm; slice thickness/slice spacing 4 mm; acquisition (reconstruction) matrix 256×224×40 (256×256×80); parallel imaging (ARC) factor 2; echo train length 120; echo-spacing ~5 ms; receiver bandwidth ±62.5 kHz; acquisition time 31 breaths (~3 min).

R_2^* mapping of the abdomen was performed in coronal orientation using a multi-echo gradient-echo sequence and 12 echo times between 2.3 and 36.2 ms with 3.1 ms echo spacing. Other parameters were as follows: TR 110 ms; flip angle 30°; FoV 40 cm × 40 cm; slice thickness 4 mm; acquisition matrix 256×224; receiver bandwidth ±62.5 kHz; parallel

Copyright: © 2022 by the authors. Licensee MDPI, Basel, Switzerland. This article is an open access article distributed under the terms and conditions of the Creative Commons Attribution (CC BY) license (https://creativecommons.org/licenses/by/4.0/).

imaging (ASSET) factor 1.5. Two slices were acquired in each 17-second breath-hold, and multiple breath-holds were used to cover the entire tumor.

Diffusion weighted imaging (DWI) was acquired in coronal orientation using a respiratory navigator-triggered dual spin-echo echo-planar imaging sequence and b-values of 0, 10, 20, 30, 100, 300, 500, 700 and 900 s/mm^2 with: echo time (TE) ~80 ms; TR 1 respiratory cycle; FoV 28.8 cm × 28.8 cm; slice thickness 4 mm; acquisition matrix 96×96; 2 averages for b-values <100 s/mm^2 and 4 averages for higher b-values; receiver bandwidth ±111 kHz; parallel imaging (ASSET: Array coil Spatial Sensitivity Encod-ing) factor 2; 3 directions averaged to form trace-weighted image; acquisition time 91 breaths (~10 min). Saturation bands were used to reduce signals from outside the volume of interest.

Dynamic Contrast-Enhanced MRI (DCE-MRI) imaging data were acquired using a Dixon imaging (LAVA-Flex) sequence in coronal orientation with the following parameters: TE 1.1/2.2 ms; TR 3.8 ms; flip angle 18°; FoV 40 cm × 40 cm; slice thickness 4 mm; acquisition (reconstruction) matrix 196×172×48 (256×256×96); receiver bandwidth ±143 kHz; parallel imaging (ARC: Autocalibrating Reconstruction for Cartesian imaging) factors 2×1.5; temporal resolution 13 s (consisting of ~7 s to acquire the images and 6 s pause for the patient to breathe); and 44 dynamic phases. In each case, the total scan time was approximately 9.5 min. Gd-DOTA (0.1 mmol/kg, Dotarem, Guerbet, Paris, France) was administered intravenously during the dynamic series at 2.5 ml/s followed by a 25 ml saline flush injected at the same rate. The injection commenced 39 s after the start of the scan. In the participant with a tumor thrombus extending into the intrahepatic inferior vena cava, angiographic triggering of the acquisition was used, and a dynamic multiphase contrast-enhanced MRI acquired. Because of the reduced temporal resolution, no quantitative modelling was performed for this participant.

The DCE-MRI acquisition was preceded by the acquisition of T_1 mapping data using a multiple-flip-angle technique (flip angles 2°, 3°, 5°, 8°, 14°); parallel imaging (ARC) factors 1.2×1.0; scan time 16 s (breath-hold) for each flip angle; other parameters as for the DCE-MRI series. B1 mapping was performed to match the DCE-MRI volume using the Bloch-Siegert method with TE 13 ms; TR 31 ms; nominal flip angle 20°; acquisition matrix 128×128; FoV 40×40 cm^2; slice thickness 12 cm; receiver bandwidth ±15.6 kHz. The DCE-MRI was processed using the extended Tofts model with a model arterial input function [1,2].

Proton Image Processing

Analysis of the intravoxel incoherent motion (IVIM) DWI data was performed using software implemented in MATLAB (The Mathworks, Natick, MA) developed by GE (GE Healthcare, Chicago IL, USA) for motion correction and developed in house for calculation of the diffusivity and perfusion fraction maps. Affine motion correction was applied across all b-values. The diffusivity D and perfusion fraction f_p were then estimated voxelwise by nonlinear fits to the multi-b-value diffusion images.

In-house software developed in MATLAB was used to generate R_2* and T_2* maps from the multi-echo gradient images. The data were fitted pixelwise to a mono-exponential decay using the nonlinear Levenberg–Marquardt algorithm, and using a log-linear approximation to compute the initial values for the fits.

The T_1 mapping and DCE-MRI data were processed in MIStar (Apollo Medical Imaging Technology, Melbourne, Australia). The T_1 mapping and DCE datasets were coregistered both within and between the datasets to remove spatial misregistration due to motion with custom software developed by GE (GE Healthcare, Chicago IL, USA). Together they were used to calculate dynamic maps of gadolinium concentration which was fitted using the Tofts model 1 with the Fritz-Hansen blood-sampled curve appended by the Weinmann wash-out as arterial input function to calculate maps of the transfer constant K^{trans} and the time to maximum contrast enhancement [2].

To allow propagation of regions of interest between sequences of a single patient, all images of a patient underwent rigid registration to the T_2w images using ITK SNAP (v3.6,

University of Pennsylvania) [3]. The tumor was outlined on the T2w HyperCube sequence on all tumor containing slices in OsiriX (Pixmeo SARL, Switzerland) and regions of interest were propagated to all other sequences. Additionally, cylindrical ROIs for individual biopsies were drawn around the location of the biopsy on the T1w LavaFlex sequence. A 3D-printed tumor mold as described below was used for the accurate co-registration of tissue samples and imaging. Voxelwise intensity values were exported using the JSON (JavaScript object notification) format and masks were created for all DCE parameters to mask out voxels with a poor fit defined as a goodness of fit value below 75%.

Supplementary References S1

1. Tofts, P.S.; Brix, G.; Buckley, D.L.; Evelhoch, J.L.; Henderson, E.; Knopp, M. V.; Larsson, H.B.W.; Lee, T.Y.; Mayr, N.A.; Parker, G.J.M.; et al. Estimating kinetic parameters from dynamic contrast-enhanced T1- weighted MRI of a diffusable tracer: Standardized quantities and symbols. *J. Magn. Reson. Imaging* **1999**, *10*, 223–232, https://doi:10.1002/(SICI)1522-2586(199909)10:3<223::AID-JMRI2>3.0.CO;2-S.
2. Fritz-Hansen, T.; Rostrup, E.; Larsson, H.B.W.; Søndergaard, L.; Ring, P.; Henriksen, O. Measurement of the arterial concentration of Gd-DTPA using MRI: A step toward quantitative perfusion imaging. *Magn. Reson. Med.* **1996**, *36*, 225–231, https://doi:10.1002/mrm.1910360209.
3. Yushkevich, P.A.; Piven, J.; Hazlett, H.C.; Smith, R.G.; Ho, S.; Gee, J.C.; Gerig, G. User-guided 3D active contour segmentation of anatomical structures: Significantly improved efficiency and reliability. *Neuroimage* **2006**, *31*, 1116–1128, https://doi:10.1016/j.neuroimage.2006.01.015.

Supplementary Figures

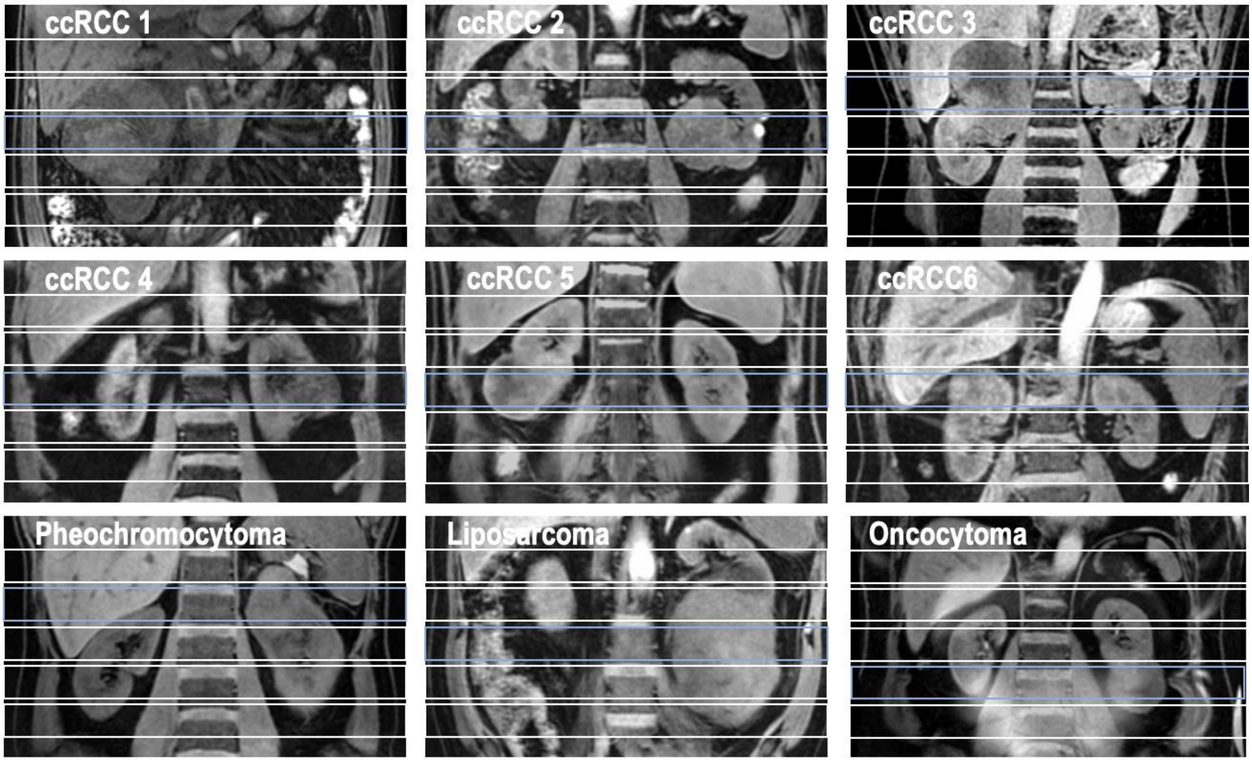

Figure S1. Positioning of the slices for the acquisition of the HP-^{13}C-pyruvate MRI. The blue slice indicates the largest cross-section of the tumor which is also shown in Figures 1, S4 and S5.

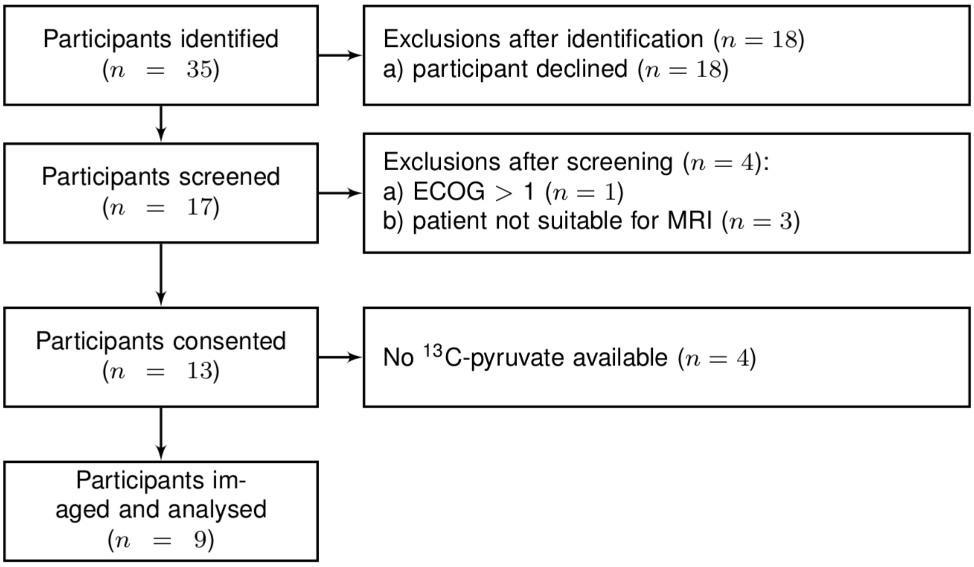

Figure S2. Patient flow diagram.

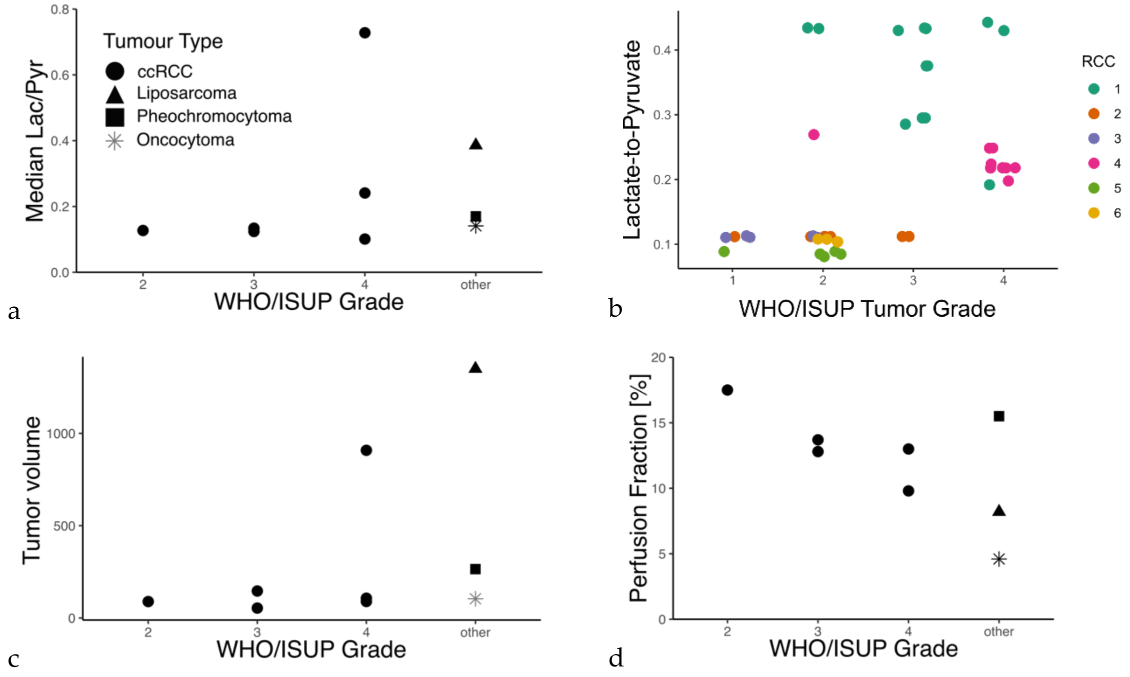

Figure S3. In contrast to the median k_{PL}, the median lactate-to-pyruvate ratio for patients (**a**), the lactate-to-pyruvate ratio for individual biopsies (**b**), and tumor volume (**c**) were not associated with ISUP/WHO grade in ccRCC. However, at the single biopsy level, an increasing lactate-to-pyruvate ratio was associated with a higher tumor grade of the corresponding tissue sample. (**d**) Increasing tumor grade was also associated with a decreasing perfusion fraction. The pheochromocytoma showed a high perfusion fraction on IVIM-DWI while the intrarenal liposarcoma showed the lowest perfusion fraction. The oncocytoma showed the lowest perfusion fraction.

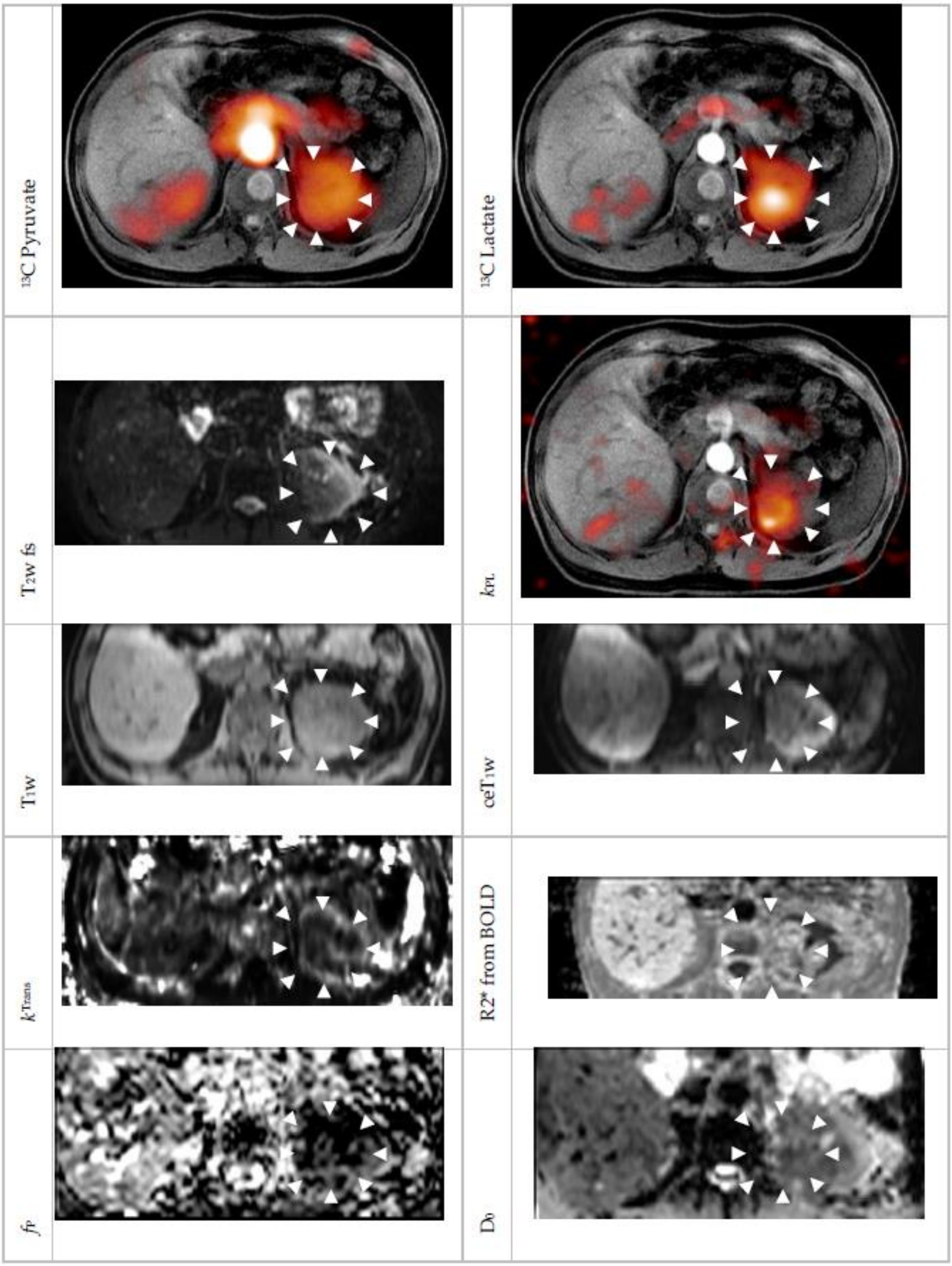

Figure S4. Representative example images of a patient with grade IV ccRCC (Patient ccRCC 4). ^{13}C-pyruvate and ^{13}C-lactate signals are summed over the entire acquisition time course and are displayed as arbitrary units. T_1w images, contrast enhanced (ce) T_1w images, K^{trans} maps, T_2w fat-suppressed (fs) images, R_2* maps, the D_0 maps equivalent to the ADC, and the perfusion fraction (f_p) maps from IVIM, have been acquired coronally and were re-formatted in the axial plane corresponding to the hyperpolarized ^{13}C pyruvate images to facilitate comparison. Arrows indicate the border of the tumor. The k_{PL} is displayed between 0 and 0.02. The D_0 map is displayed between 0 and 3.0×10^{-3} mm/s^2.

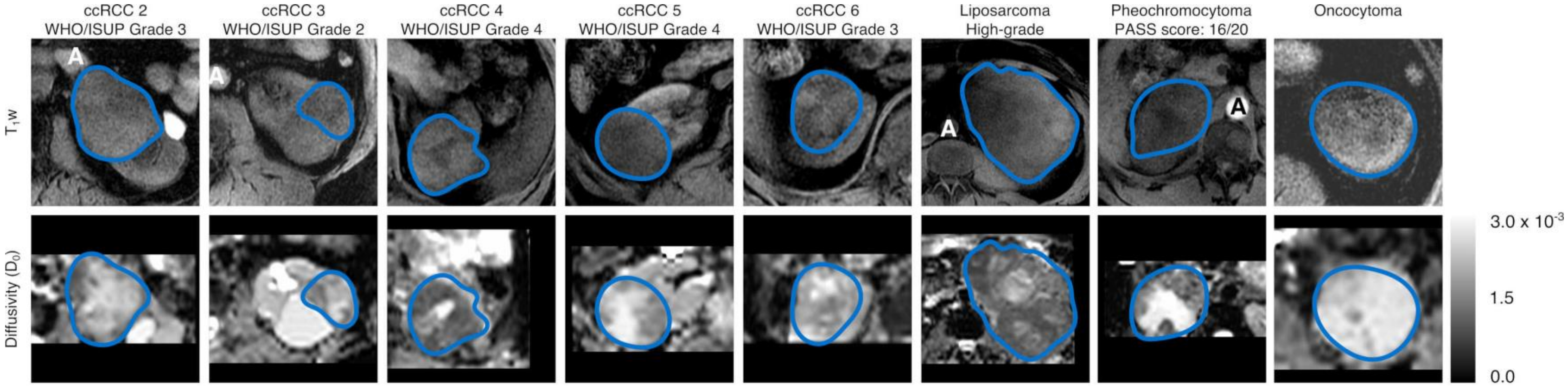

Figure S5. Diffusivity (D_0) maps corresponding to the metabolic images presented in Figure 1 of the manuscript.

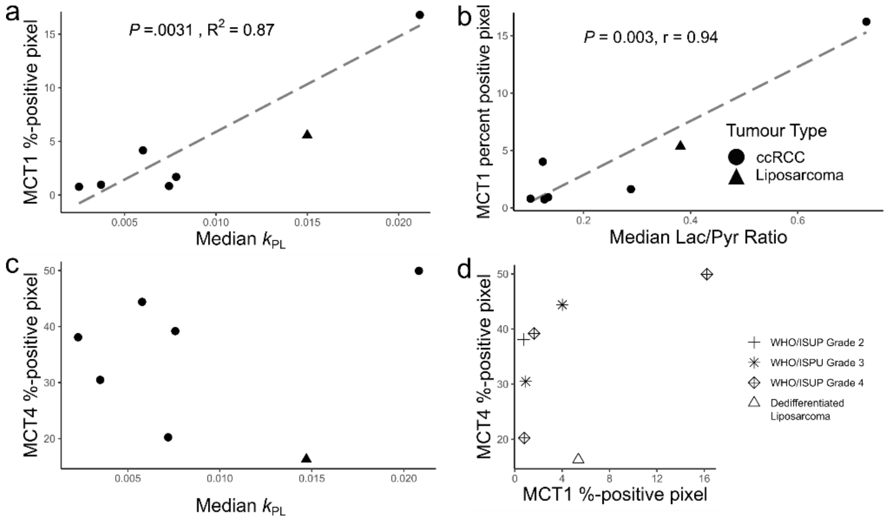

Figure S6. Immunohistochemistry for the monocarboxylate transporters MCT1 and MCT4 from biopsy samples. The percentage of positive pixels was averaged across all biopsies from one patient. (a) Correlation between tumor MCT1 expression and k_{PL} on hyperpolarized HP-^{13}C-MRI. (b) Correlation between tumor MCT1 expression and pyruvate-to-lactate ratio on hyperpolarized ^{13}C-pyruvate MRI. (c) No significant correlation was found between tumor MCT4 expression and k_{PL} on hyperpolarized ^{13}C-pyruvate MRI. (d) Neither MCT1 expression nor MCT4 expression was associated with tumor grade.

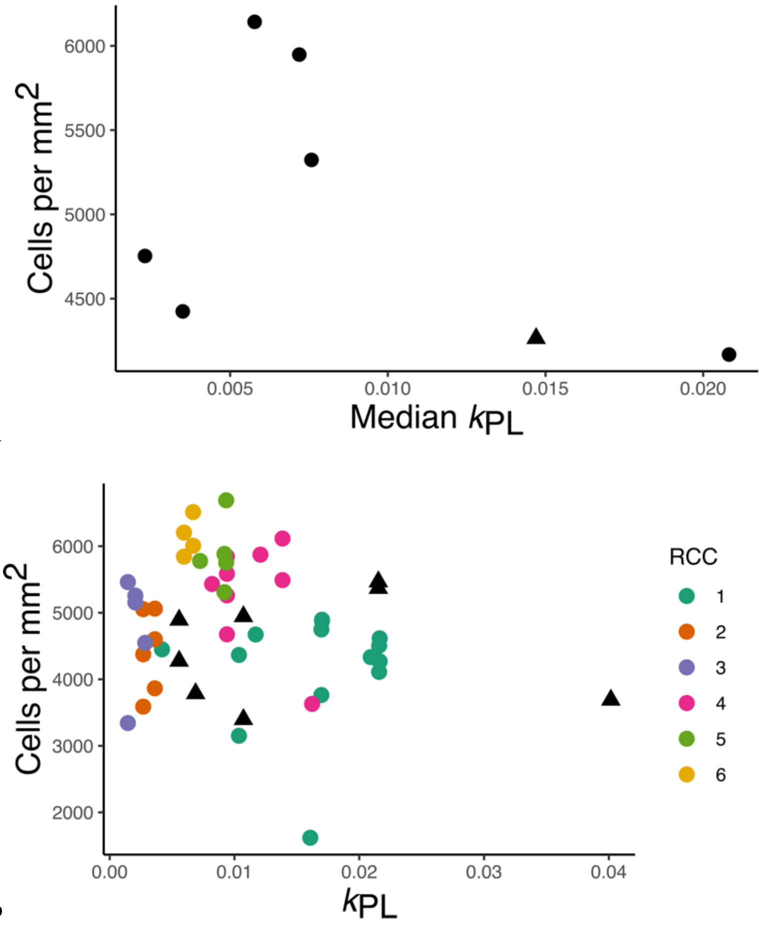

a

b

Figure S7. The k_{PL} was not associated with the histologically determined cell density, neither for the whole tumor nor for individual biopsies.

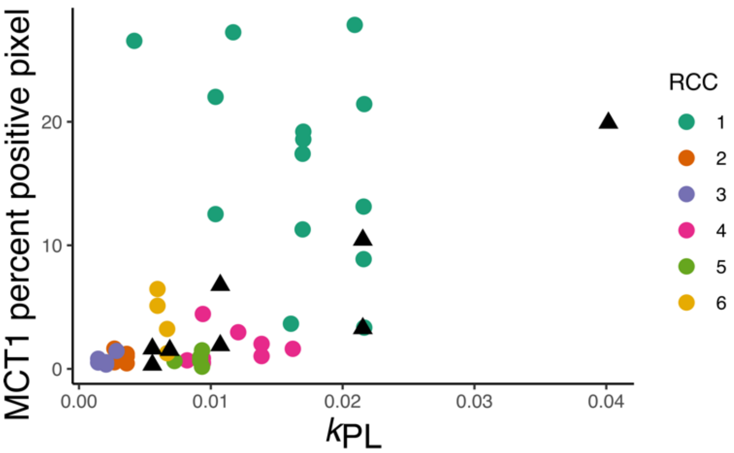

Figure S8. A linear mixed model was employed for modelling the effect of k_{PL} on the expression of MCT1 on the individual biopsy level while taking the patient dependence into account as a random effect. It should be noted that the variance of the explanatory variable was not independent of the random effect. The variance in MCT1 expression was borderline significantly associated with the median k_{PL} (p = 0.052). These analyses have shown that the variation in k_{PL} can be interpreted as correlated with the random effect (patient) or the fixed effect (MCT1).

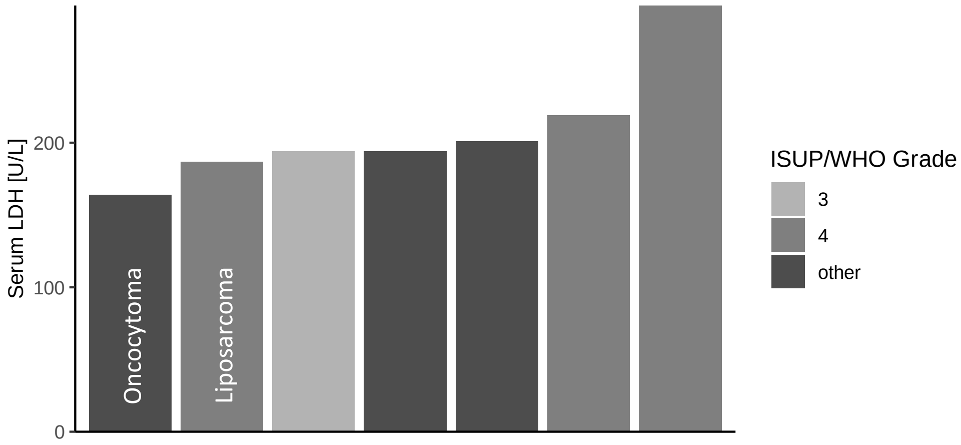

Figure S9. No correlation between tumor aggressiveness and serum LDH concentrations was observed.

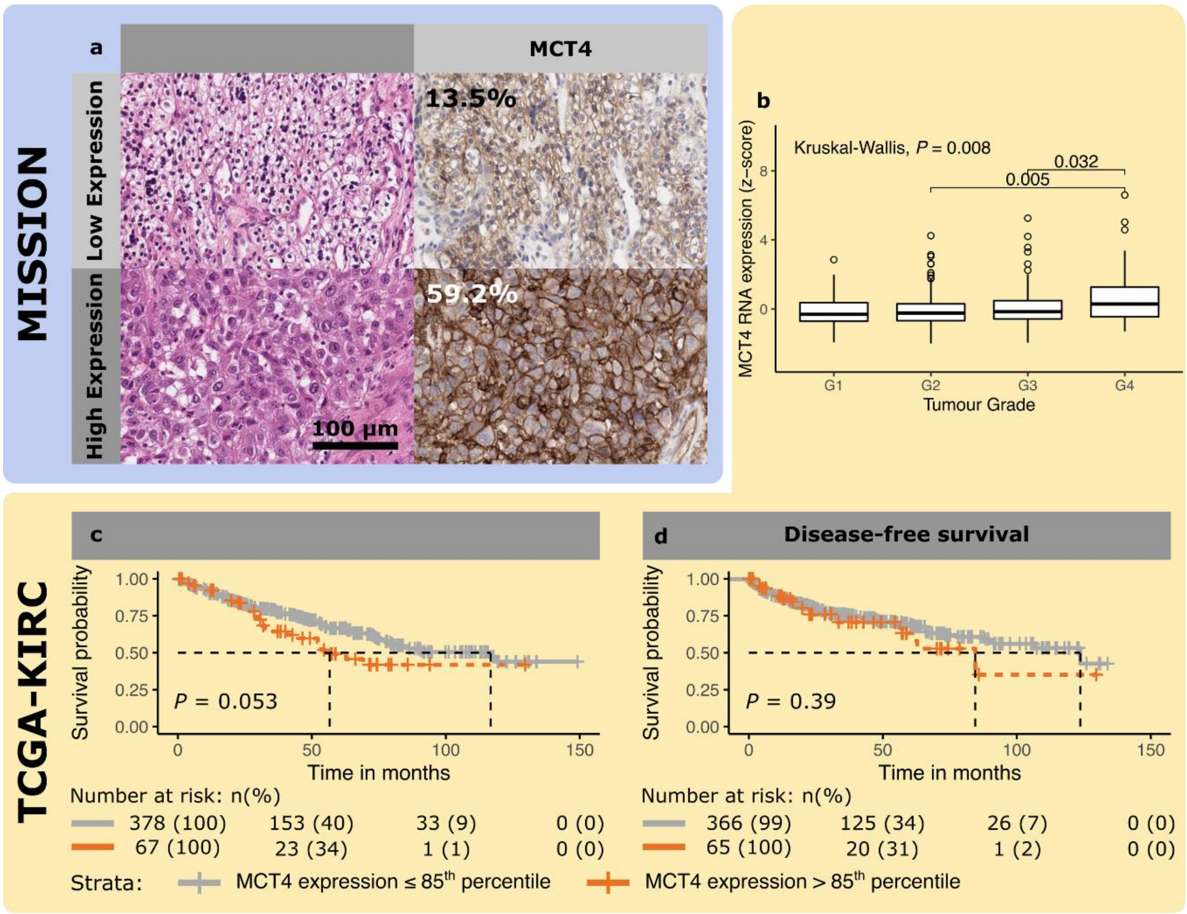

Figure S10. Representative micrographs of a clear cell renal cell carcinoma with high and low expression of MCT4. (**a**) The left panels show a hematoxylin and eosin stain and the right panels the corresponding immunohistochemical stain for MCT4 on an adjacent tissue slice. (**b**) Box plot comparing z-transformed MCT4 expression as a function of histological tumor grade in the MISSION dataset (blue). (**c**) Kaplan-Meier plots for the association of MCT4 expression with overall and (**d**) progression/recurrence-free survival using an expression cut-off at the 85th percentile in the TCGA KIRC (yellow). MCT4 expression was only significantly associated with overall survival.

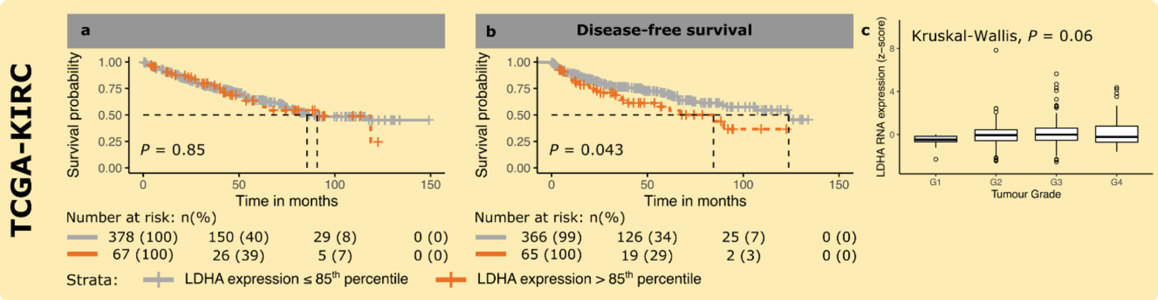

Figure S11. Kaplan-Meier plots for the association of LDHA expression with (**a**) overall and (**b**) progression/recurrence-free survival using an expression cut-off at the 85th percentile. LDHA expression was only significantly associated with progression/recurrence-free survival. (c) Box plot comparing z-transformed MCT4 expression as a function of histological tumor grade. All data were derived from the TCGA KIRC dataset.

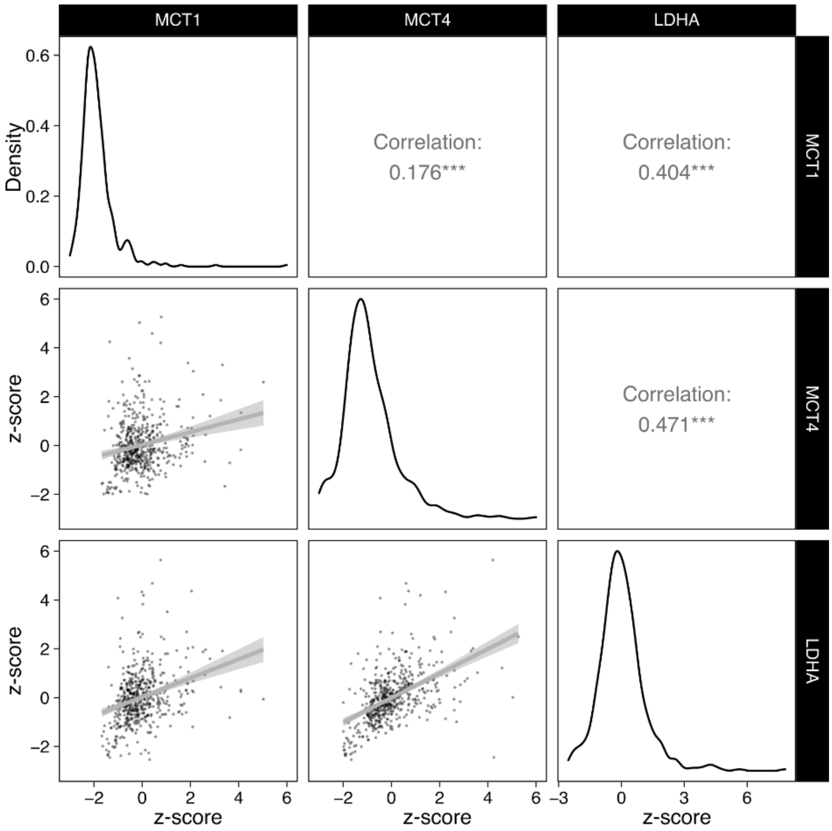

Figure S12. Correlation between gene expression parameters in the TCGA-KIRC dataset. Gene expressions were z-score transformed. *** $p < 0.001$. Grey lines and sector show the regression line and the 95%-confidence interval.

Supplementary Tables

Table S1. Proton MR parameters for all patients except ccRCC 1.

Sequence	TE [ms]	TR [ms]	Flip Angle [deg]	Matrix size	Field of View [mm]	Slice Thickness [mm]	Orientation	Type	Parallel imaging factor	Comments
T_1w LavaFlex	1.1 / 2.2	3.8	10	224 × 224	400 × 400	4	Coronal	3D	1.5 (ARC)	Single BH
T_2w FOCUS CUBE	100	respiratory triggered	90	256 × 224	360 × 360	4	Coronal	3D	2.0 (ARC)	RT, ETL: 120
T_2* / R_2* mapping	2.3–36.2 (12 echoes, 3.1 ms spacing)	110	30	256 × 224	400 × 400	4	Coronal	2D	2.0 (ASSET)	Multiple BH, ETL: 12
IVIM-DWI	80	respiratory triggered	90	96 × 96	288 × 288	4	Coronal	2D	1.5 (ASSET)	b-values: 0, 10, 20, 30, 50, 100, 300, 500, 700, 900 s/mm^2
B_1 mapping	13	31		128 × 128	400 × 400	12	Coronal	2D		Bloch-Siegert method
T_1 mapping	1.1 / 2.2	3.8	2, 3, 5, 8, 14	196 × 172	400 × 400	4	Coronal	3D	1.2 × 1.0 (ARC)	Single BH
DCE-MRI	1.1 / 2.2	3.8	18	196 × 172	400 × 400	4	Coronal	3D	2.0 × 1.5 (ARC)	0.1 ml of 1M Gadobutrol i.v. temporal resolution: 13s

ARC: Autocalibrating reconstruction for cartesian imaging, ASSET: Array coil spatial sensitivity encoding BH: Breath hold, ETL: Echo train length, RT: Respiratory triggered.

Table S2. Proton MR parameters for ccRCC 1.

Sequence	TE [ms]	TR [ms]	Flip Angle [deg]	Matrix size	Field of View [mm]	Slice Thickness [mm]	Orientation	Type	Comments
T_1w LavaFlex	1.1 / 2.2	3.8	10	224 x 224	400 x 400	2	Coronal	3D	Single BH
T_2w FOCUS CUBE	65	respiratory triggered	90	192x 192	380 x 380	2	Coronal	3D	RT, ETL: 120
T_2* / R_2* mapping	2.3–36.2 (12 echoes, 3.1 ms spacing)	110	30	256 x 224	400 x 400	4	Coronal	2D	Multiple BH, ETL: 12
DWI	49	2000	90	128 x 80	380 x 380	8	Axial	2D	RT
Multi-phase ceMRI	1.9	4.6	12	320 x 224	400 x 400	4.6	Coronal	3D	Multiple BH, 8 phoases in 5 minutes

BH: Breath hold, ceMRI> contrast/enhanced MRI, ETL: Echo train length, RT: Respiratory triggered.

Table S3. ^{13}C pyruvate preparation QC parameters.

Quality control parameter	Mean ± S.D.
Polarization [%]	24.5 ± 7.8
pH	7.7 ± 0.3
EPA concentration [µM]	0.8 ± 0.4
Temperature [°C]	33.6 ± 1.0
Pyruvate concentration [mM]	261.6 ± 7.2
Injected Volume [mL]	34.9 ± 3.6
Time from dissolution to injection [%]	60.3 ±3.9

S.D.: standard deviation, EPA: electron paramagnetic agent.

Table S4. Multivariate logistic regression for progression-free survival in the TCGA KIRC cohort.

			95% Confidence Interval	
Covariates	*p* value	HR	Lower	Upper
Age	0.10	1.020	0.996	1.044
Female Sex	0.63	0.875	0.505	1.514
Lymph nodes	0.013	3.048	1.256	7.399
Metastasis	<0.001	5.812	3.323	10.166

Size	0.52	1.150	0.752	1.759
Grade				
Grade 1	0.49	2.135	0.245	18.606
Grade 2	0.038	0.445	0.208	0.957
Grade 3	0.37	0.722	0.352	1.481
MCT1	0.11	1.178	0.960	1.45
MCT4	0.85	1.024	0.790	1.327
LDHA	0.91	0.988	0.792	1.232

Concordance index = 0.79.
